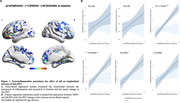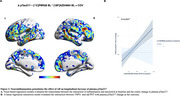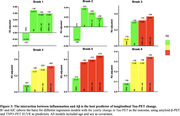# Neuroinflammation potentiates the effect of amyloid‐β on longitudinal tau changes

**DOI:** 10.1002/alz.094105

**Published:** 2025-01-09

**Authors:** Nesrine Rahmouni, Yi‐Ting Wang, Joseph Therriault, Stijn Servaes, Cécile Tissot, Arthur C. Macedo, Jaime Fernandez Arias, Seyyed Ali Hosseini, Jenna Stevenson, Peter Kunach, Brandon J Hall, Lydia Trudel, Serge Gauthier, Andreas Jeromin, Henrik Zetterberg, Kaj Blennow, Eduardo R. Zimmer, Nicholas J. Ashton, Andrea L. Benedet, Tharick Ali Pascoal, Pedro Rosa‐Neto

**Affiliations:** ^1^ Translational Neuroimaging Laboratory, The McGill University Research Centre for Studies in Aging, Montréal, QC Canada; ^2^ Lawrence Berkeley National Laboratory, Berkeley, CA USA; ^3^ McGill University, Montreal, QC Canada; ^4^ UTSouthwestern, Dallas, TX USA; ^5^ Translational Neuroimaging Laboratory, The McGill University Research Centre for Studies in Aging, Montreal, QC Canada; ^6^ ALZpath, Inc., Carlsbad, CA USA; ^7^ University of Gothenburg, Mölndal, Gothenburg Sweden; ^8^ University of Gothenburg, Gothenburg, Västergötland Sweden; ^9^ Federal University of Rio Grande do Sul (UFRGS), Porto Alegre, RS Brazil; ^10^ University of Gothenburg, Gothenburg Sweden; ^11^ University of Pittsburgh, Pittsburgh, PA USA

## Abstract

**Background:**

It has been proposed that microglia release of proinflammatory factors reactive to amyloid plaques constitutes an early event leading to tau pathology. Here, we assessed how the rate of progression of tau‐PET and the rate of change in plasma pTau217 are affected by baseline levels of amyloid‐ß and neuroinflammation.

**Methods:**

We included 93 individuals from TRIAD cohort: 11 young individuals, 57 cognitively unimpaired elderlies, 15 with mild cognitive impairment and 10 individuals with Alzheimer’s Disease. Neuroinflammation, tau tangle and amyloid‐ß (Aß) deposition were assessed via [11C]PBR28‐PET, [18F]MK6240‐PET and [18F]AZD4694‐PET, respectively. Individuals also had plasma pTau217 quantified using the Alzpath assay. [11C]PBR28‐PET positivity was defined as a SUVR of 2.5SD above the mean of the young individuals in the precuneus and posterior cingulate. Voxel‐based regression models evaluated the interaction of TSPO‐ and Aß‐PET with annual change in tau‐PET and plasma pTau217. The effect of the interaction on tau‐PET was also evaluated at the region‐of‐interest level using Braak regions. All models were corrected for age, sex and RFT corrected. Comparison of models was made using the Akaike Information Criterion (AIC) and the models’ goodness‐of‐fit was evaluated using R2 analyses. Models used [18F]MK6240‐PET SUVR yearly change as the outcome and used as predictors [11C]PBR28‐PET SUVR in the precuneus and posterior cingulate and [18F]AZD4694‐PET SUVR in the neocortical region.

**Results:**

Positive associations were found between the interaction of Aß‐ and TSPO‐PET at baseline predicting the yearly change in tau‐PET (Figure 1A). At the ROI level, the interaction significantly predicts tau load in Braak regions III‐IV in [11C]PBR28 positive individuals (Figure 1B). Positive associations were also found between the interaction of Aß‐ and TSPO‐PET at baseline predicting the yearly change in plasma pTau217 (Figure 2A), which is driven by [11C]PBR28 positive individuals (Figure 2B). In addition, the interaction between Aß‐ and TSPO‐PET predicting the yearly change in tau‐PET is the model with the highest R2 and the lowest AIC values in Braak regions III‐IV‐V‐VI (Figure 3).

**Conclusion:**

These results support the hypothesis that microglial activation potentiates the effect of Aß plaques on tau tangle progression and the pTau217 increases across the aging and AD spectrum.